# Monozygotic triplets with juvenile-onset autoimmunity and 18p microdeletion involving *PTPRM*

**DOI:** 10.3389/fgene.2024.1437566

**Published:** 2024-09-18

**Authors:** Morten Krogh Herlin, Jens Magnus Bernth Jensen, Lotte Andreasen, Mikkel Steen Petersen, Jonas Lønskov, Mette Bendixen Thorup, Niels Birkebæk, Trine H. Mogensen, Troels Herlin, Bent Deleuran

**Affiliations:** ^1^ Department of Clinical Genetics, Aarhus University Hospital, Aarhus, Denmark; ^2^ Department of Clinical Immunology, Aarhus University Hospital, Aarhus, Denmark; ^3^ Department of Molecular Medicine, Aarhus University Hospital, Aarhus, Denmark; ^4^ Department of Biomedicine, Aarhus University, Aarhus, Denmark; ^5^ Department of Radiology, Aarhus University Hospital, Aarhus, Denmark; ^6^ Department of Pediatrics and Adolescent Medicine, Aarhus University Hospital, Aarhus, Denmark; ^7^ Department of Clinical Medicine, Aarhus University, Aarhus, Denmark; ^8^ Department of Infectious Diseases, Aarhus University Hospital, Aarhus, Denmark; ^9^ Department of Rheumatology, Aarhus University Hospital, Aarhus, Denmark

**Keywords:** 18p deletion, autoimmunity, cytogenetics, PTPRM, STAT3 transcription factor, Th17 cells

## Abstract

Abnormal gene dosage from copy number variants has been associated with susceptibility to autoimmune disease. This includes 18p deletion syndrome, a chromosomal disorder with an estimated prevalence of 1 in 50,000 characterized by intellectual disability, facial dysmorphology, and brain abnormalities. The underlying causes for autoimmune manifestations associated with 18p deletions, however, remain unknown. Our objective was to investigate a distinctive case involving monozygotic triplets concordant for developmental delay, white matter abnormalities, and autoimmunity, specifically juvenile-onset Graves’ thyroiditis. By chromosomal microarray analysis and whole genome sequencing, we found the triplets to carry a *de novo* interstitial 5.9 Mb deletion of chromosome 18p11.31p11.21 spanning 19 protein-coding genes. We conducted a literature review to pinpoint genes affected by the deletion that could be associated with immune dysregulation and identified PTPRM as a potential candidate. Through dephosphorylation, PTPRM serves as a negative regulator of STAT3, a key factor in the generation of Th17 cells and the onset of specific autoimmune manifestations. We hypothesized that PTPRM hemizygosity results in increased STAT3 activation. We therefore performed assays investigating PTPRM expression, STAT3 phosphorylation, Th1/Th2/Th17 cell fractions, Treg cells, and overall immunophenotype, and in support of the hypothesis, our investigations showed an increase in cells with phosphorylated STAT3 and higher levels of Th17 cells in the triplets. We propose that PTPRM hemizygosity can serve as a contributing factor to autoimmune susceptibility in 18p deletion syndrome. If confirmed in unrelated 18p/PTPRM deletion patients, this susceptibility could potentially be treated by targeted inhibition of IL-17.

## 1 Introduction

Abnormal gene dosage resulting from various chromosomal imbalances has been associated with susceptibility to autoimmune diseases and these aberrations are important for understanding the complex genetic architecture of autoimmunity (Kyritsi and Kanaka-Gantenbein, 2020). Monosomy 18p or 18p deletion syndrome is a chromosomal disorder with an estimated prevalence of 1 in 50,000 live births (Turleau, 2008). It was first described in 1963 by the French geneticist Jean de Grouchy (de Grouchy, 1963). Most reported deletions are terminal spanning large proportions of the 18p arm, whereas interstitial microdeletions are rare (Hasi-Zogaj et al., 2015).

Typical features of 18p deletion syndrome include mild to moderate developmental delay and subtle facial dysmorphology. Abnormal magnetic resonance imaging (MRI) findings in the brain are common, especially white matter abnormalities (Hasi-Zogaj et al., 2015). More severe brain abnormalities include holoprosencephaly, which has been linked to *TGIF1* hemizygosity (Hasi-Zogaj et al., 2015). Some patients present with various autoimmune diseases (Turleau, 2008; Hasi-Zogaj et al., 2015; Sebold et al., 2015; McGoey et al., 2011), including thyroid disease, rheumatoid arthritis, celiac disease, alopecia, and others. Autoimmune thyroid disease reported in 18p deletion syndrome includes both Hashimoto’s (McGoey et al., 2011; Bühler et al., 1964; Ruvalcaba, 1970; Gluckman, 1977) and Graves’ (Jones and Carey, 1982; Dharmaraj and Grueters, 2006) thyroiditis. *PTPN2* has been considered a candidate gene for autoimmune susceptibility but is not included in the 18p chromosomal region associated with autoimmune disorders (Supplementary Figure S1) (Hasi-Zogaj et al., 2015; Cody et al., 2018). The understanding of autoimmunity in 18p deletion syndrome therefore remains elusive.

In this report, we present a distinctive case involving monozygotic triplets all with intellectual disability, concordance for childhood-onset Graves’ thyroiditis, and a *de novo* interstitial 18p microdeletion. Furthermore, we explore the potential role of one of the deleted genes, *PTPRM*, in relation to their immune-related conditions.

## 2 Methods

### 2.1 Case description

**FIGURE 1:**
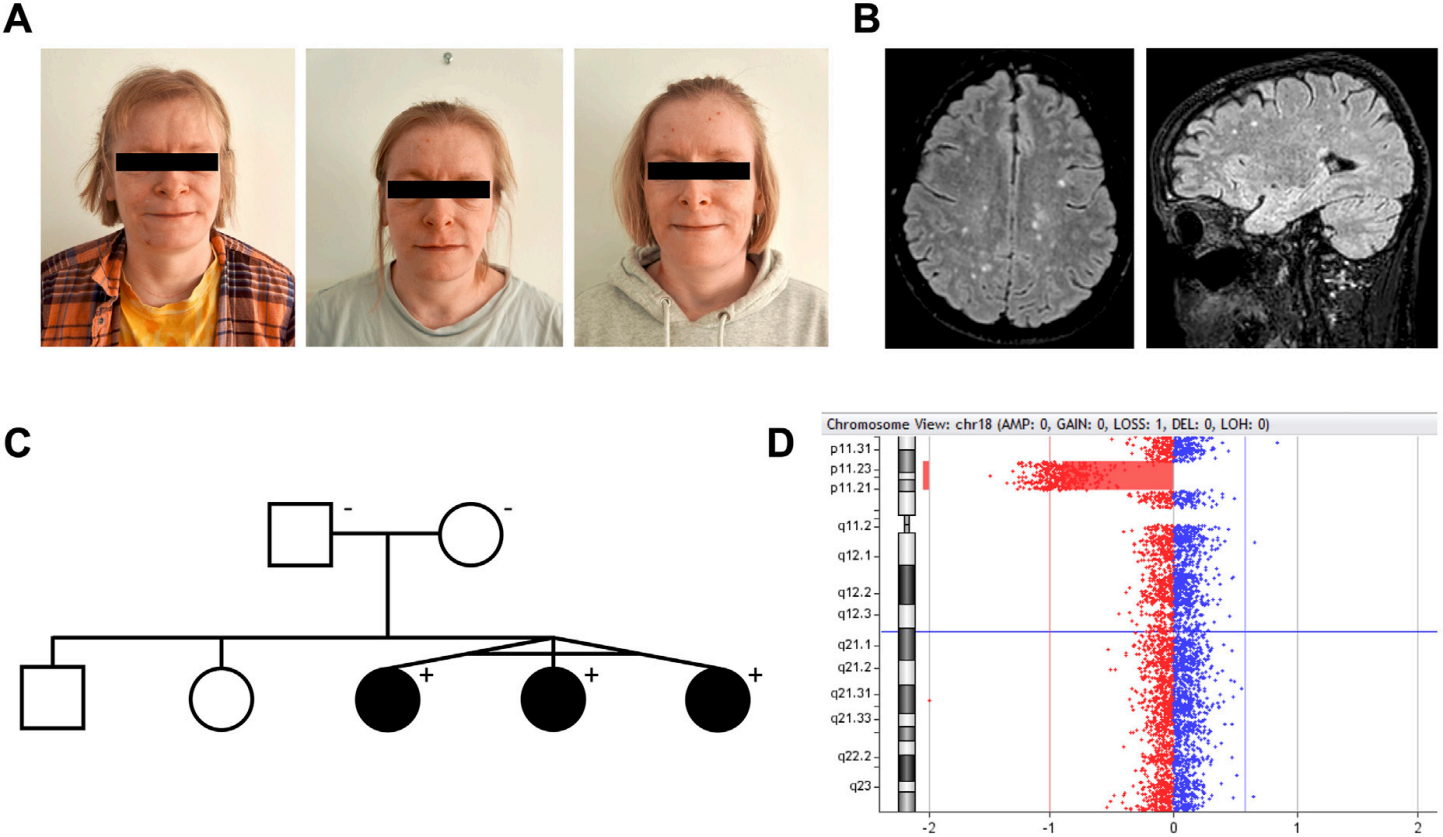
Interstitial 18p microdeletion identified in monozygotic triplets with juvenile-onset autoimmunity, intellectual disability, and white matter lesions. **(A)** Monozygotic triplets, 37 years old [left to right: triplet A–C]. **(B)** Brain MRI of triplet A. Axial and sagittal FLAIR (1.5 T) showing multiple small, primarily subcortical white matter lesions, as frequently reported in 18p deletion syndrome patients. All triplets had these lesions, which were found stable over time. **(C)** Pedigree of the family. The deletion was not present in the healthy parents. **(D)** Visualization of the 18p11.31p11.21 microdeletion in CytoGenomics.

Monozygotic female triplets (Figures 1A–C), 37 years old, were referred for clinical genetic evaluation due to a complex history of immune-related disorders, mild to moderate intellectual disability, and psychomotor developmental delay. The triplets were delivered by cesarean section after preterm premature rupture of membranes at gestational age 34 + 0. At the age of 6 years, standard chromosomal analysis was carried out reporting normal karyotypes (46,XX) in all three individuals. Case descriptions with detailed summaries of clinical features are provided in Table 1.

**TABLE 1:** Clinical history and presentation of the triplets with 18p11 microdeletion.

	Triplet A	Triplet B	Triplet C
Age, years Height, cm Weight, kg	37 163 68	37 163 78	37 163 68
Gestational age Birth weight, gram	34 + 0 1994	34 + 0 1908	34 + 0 1708
Developmental milestones	2 years: walking Delayed speech development	2 years: walking Delayed speech development	2 years: walking Delayed speech development
Central nervous system	Mild to moderate ID Verbal and motor dyspraxia WM lesions Ataxia and wide-based gait	Mild to moderate ID Verbal and motor dyspraxia WM lesions Ataxia and wide-based gait	Mild to moderate ID Verbal and motor dyspraxia WM lesions Ataxia and wide-based gait
Facial dysmorphology	Drooping eyelids Hypertelorism Low-hanging columella Anteverted nares Thin upper-lip vermillion Protruding philtrum	Drooping eyelids Hypertelorism Low-hanging columella Anteverted nares Thin upper-lip vermillion Exophthalmos following Graves’ ophthalmopathy	Low-hanging columella Anteverted nares Thin upper-lip vermillion Protruding philtrum
Respiratory	17 years: small lung infiltrations	—	17 years: small lung infiltrations
Cardiovascular	Cardiomegaly Normal cardiac function	Cardiomegaly Left ventricular hypertrophy/dilation Normal cardiac function	Cardiomegaly Left ventricular dilation Normal cardiac function 35 years: hospitalized with infectious endocarditis
Thyroid manifestations	7.7 years: Graves’ disease, 9 years: PTU-induced lupus-like reaction [see Ref. (Herlin et al., 2002)] 17 years: Thyroidectomy + levothyroxine substitution	7.5 years: Graves’ disease 9 years: PTU-induced lupus-like reaction [see Ref. (Herlin et al., 2002)] 17 years: Thyroidectomy + levothyroxine substitution	9.5 years: Graves’ disease 17 years: Thyroidectomy + levothyroxine substitution
Skin	11 years: pruritus and neurotic excoriations 20 years: chronic urticaria Raynaud’s phenomenon	18 years: morphea Raynaud’s phenomenon	17 years: pruritus and neurotic excoriations 29 years: chronic urticaria 30 years: severe angioedema Raynaud’s phenomenon
Gynecology/reproductive endocrinology	17 years: menarche 17 years: hirsute hair growth, normal androgens 30 years: benign ovary cyst	16 years: menarche 20 years: hirsutism, low SHBG and high FAI 34 years: PCOS, oligomenorrhea, surgical removal of one cyst	NS
Urogenital	10 years: diurnal enuresis (urge urine incontinence)	10 years: diurnal enuresis (urge urine incontinence)	14 years: diurnal enuresis (urge urine incontinence)
Skeletal	Accessory bone by the left hamate	Functional LLD: 2 cm Scoliosis	—
Psychiatry	25 years: OCD	—	18 years: compulsions, not formally diagnosed with OCD
Immunoglobulin A	Normal	NS	Normal

Abbreviations: FAI, free androgen index; ID, intellectual disability; LLD, leg length discrepancy; NS, not stated; OCD, obsessive-compulsive disorder; PCOS, polycystic ovary syndrome; PTU, propylthiouracil; SHBG, sex-hormone binding globulin; WM, white matter.

At the age of 7 years, two of the triplets (A + B) presented with signs of thyrotoxicosis within a few months. A diagnosis of Graves’ autoimmune thyroiditis was reached and antithyroid treatment was initiated with propylthiouracil (PTU) effectively reaching an euthyroid state. After 18 and 20 months of treatment, both triplets A and B developed lupus-like manifestations with macular exanthema and synovitis of the wrists and ankles. A drug-induced reaction was suspected and PTU was replaced by methimazole. Two years after the referral of triplet A + B, triplet C also developed signs of hyperthyroidism and was diagnosed with Graves’ thyroiditis. She was treated with methimazole and not PTU and never developed a rash. At age 17, all triplets had a thyroidectomy and subsequent levothyroxine replacement in order to obtain better control of symptoms. The PTU-induced reaction in the triplets including detailed serological analyses has previously been reported by Herlin et al. (2002).

As part of examinations during childhood, brain magnetic resonance imaging (MRI) was done in triplet A + B identifying multiple small subcortical and periventricular white matter (WM) lesions (Figure 1B), initially thought to present manifestations of systemic vasculitis following PTU exposure. However, similar WM lesions were later found in triplet C, suggesting the lesions as an inherent feature of their underlying condition rather than related to PTU exposure. During their clinical courses, several MRIs were performed showing no signs of progression of these lesions over time in all triplets. No brain malformations were observed.

Other immune-related features included the development of chronic urticaria in triplet A+ C and triplet C later developing severe angioedema. Triple A and C also had unspecific lung findings on computed tomography (CT) scans suggestive of interstitial lung disease, which together with the observed WM lesions raised suspicion of an immune-related vasculopathy. All triplets had measurements of elevated myeloperoxidase anti-neutrophil cytoplasmic antibody (MPO-ANCA) during their follow-up (triplet A, 3.7 to >100 kIU/L; triplet B, 13 kIU/L; triplet C, 12–23 kIU/L; reference level <3.5 kIU/L). None of the triplets had malignancy. Altogether, based on the history of concordant juvenile onset of Graves’ thyroiditis, PTU-induced immune reactions, chronic urticaria/angioedema, lung findings, and elevated MPO-ANCA in the monozygotic triplets with developmental delay, we hypothesized that an underlying genetic condition to cause the immune dysregulation, WM lesions, and neurological manifestations.

### 2.2 Genetic analysis

Genomic DNA was extracted from peripheral blood of the three patients and their parents. To screen for copy number variations, array-based comparative genomic hybridization (CGH) was performed for all triplets and their parents using the SurePrint G3 Human CGH microarray 4 × 180 K (Agilent Technologies). For accurate breakpoint characterization and sequence analysis for biallelic involvement of the deleted genes, we subsequently performed whole genome sequencing (WGS) (NovaSeq 6000 platform, Illumina Inc.) of triplet A. WGS data was also examined using an *in silico* gene panel of 341 genes related to cellular and innate immunity. Detailed methodological descriptions are provided in the Supplementary Material.

Variant descriptions were written following HGVS nomenclature recommendations.

### 2.3 Flow cytometry

Phosphorylation of signal transducer and activator of transcription 3 (STAT3) in interleukin-6 (IL-6) stimulated peripheral blood cells was examined by flow cytometry using intracellular staining with fluorophore-labeled phosphorylation-specific STAT3 (pY705) antibody (BD Biosciences). Flow cytometry was also used to quantify selected subsets of peripheral lymphocytes, e.g., Th17 cells and Tregs. Detailed methodological descriptions are provided in the Supplementary Material.

### 2.4 Western blot analysis of PTPRM expression

In a *post hoc* analysis, we attempted to measure protein tyrosine phosphatase receptor type M (PTPRM) protein expression in patient peripheral blood mononuclear cells (PBMCs) as well as patient serum due to low expression of PTPRM in human blood cells (EMBL-EBI Expression Atlas, https://www.ebi.ac.uk/gxa/home, data accessed 9 Nov 2023). Results were compared to parent/control samples. Detailed methodological descriptions are provided in the Supplementary Material.

### 2.5 Ethical statement

According to Danish legislation, laboratory studies by clinical indication do not require a formal ethics committee assessment. A query was sent to The Central Denmark Region Committees on Health Research Ethics (record no. 1-10-72-103-24). Written consent was obtained from the patients and their parents, including consent for publication of photographs in Figure 1A.

## 3 Results

Array CGH analysis of the triplets revealed an interstitial 5.9 Mb deletion of chromosome 18p11.31p11.21, NC_000018.10: g. (5416774_5424248)_(11251959_11286578)del (Figure 1D). The accurate breakpoints of the deletion detected by WGS analysis were NC_000018.10: g.5420142_11286542del (Supplementary Figure S2). From WGS analysis, no predicted pathogenic variants were found at the *trans* allele at 18p11.31p11.21 or within 341 genes related to cellular and innate immunity (Supplementary Table S1). Array CGH and chromosomal banding analyses of the parents were normal (Figure 1C).

The deletion included a total of 19 protein-coding genes listed in RefSeq: *EPB41L3*, *TMEM200C*, *L3MBTL4*, *ARHGAP28*, *LAMA1*, *LRRC30*, *PTPRM*, *RAB12*, *MTCL1*, *NDUFV2*, *ANKRD12*, *TWSG1*, *RALBP1*, *PPP4R1*, *RAB31*, *TXNDC2*, *VAPA*, *APCDD1*, and *PIEZO2*. The deletion is contained within the 18p region which previously has been associated with autoimmunity and partly overlaps the region associated with thyroid disease as stated in the Chromosome 18 Gene Dosage Map by Cody and colleagues (Supplementary Figure S1) (Hasi-Zogaj et al., 2015; Cody et al., 2018). This evidence together with the triplet’s concordance for juvenile-onset autoimmunity, led us to question if hemizygosity of one or more genes within the interstitial 18p deletion could explain the autoimmune susceptibility in the triplets. Established disease-associated genes within the deletion, as defined by Online Mendelian Inheritance in Man (OMIM), include *APCDD1*, *LAMA1, NDUFV2, and PIEZO2*. Isolated monoallelic deletion of these genes could not explain the observed phenotype in the triplets (details provided in the Discussion) and we therefore reviewed the available literature on the other deleted genes and speculated on possible connections to immune dysregulation, including connections to immune-related signalling pathways. From this search, we identified *PTPRM* as a potential candidate gene for autoimmunity/immune dysregulation. *PTPRM* encodes the protein tyrosine phosphatase receptor type M (PTPRM). PTPRM is reported to dephosphorylate and thereby negatively regulate the activation of STAT3 (Im et al., 2020; Song et al., 2021), a crucial transcription factor in the immune system with links to autoimmunity. We therefore hypothesized that the deletion may cause immune dysregulation through an increase in cells with phosphorylated STAT3.

Of note, the deletion also involved *MTCL1* which previously has been proposed as a candidate gene for ataxia (Satake et al., 2017; Krygier et al., 2019). All triplets had wide-based gait as well as verbal and motor dyspraxia, which could suggest some cerebellar dysfunction (Table 1).

### 3.1 *PTPRM* hemizygosity associated with increase in cells with phosphorylated STAT3 and Th17 cell preponderance

We examined their peripheral blood lymphocytes and found that total lymphocyte concentrations were slightly below the reference range while the absolute concentrations of T-, B-, and NK-cells were each within reference ranges (Supplementary Table S2). Among T-cells, we observed unremarkable distributions between T-helper cells and cytotoxic T-cells. Proportions of naïve T-cells, regulatory T-cells, and activated T-cells (HLA-DR+) were also within reference ranges. However, the triplets’ B-cells exhibited slightly reduced fractions of class-switched memory B-cells and marginal zone-like B-cells, while their fractions of plasmablasts were slightly increased.

**FIGURE 2:**
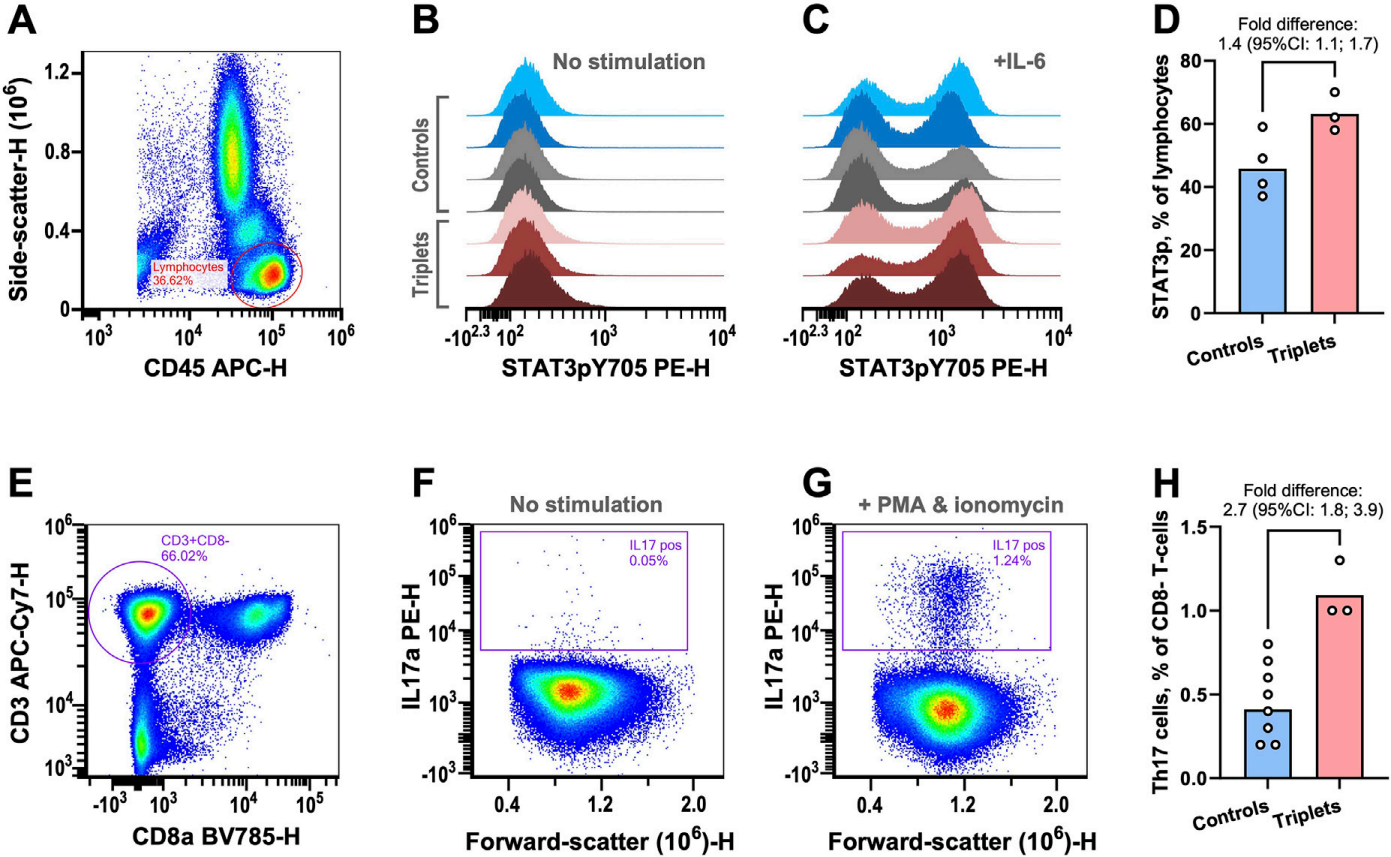
Functional immunological analyses. **(A–D)** STAT3 phosphorylation was examined in peripheral lymphocytes by flow cytometry. **(A)** Example plot showing the gating strategy for lymphocyte isolation. **(B)** STAT3 phosphorylation signal from lymphocytes without prior stimulation for healthy blood donors (blue), parents (grey), and triplets (red). **(C)** As in previous panel but with prior IL-6 stimulation. **(D)** The fractions of lymphocytes with IL6-induced STAT3 phosphorylation in controls (healthy blood donors and parents) and triplets. The columns are the geometric means of the two groups. The fold-difference of the means was estimated with 95% confidence intervals (CIs) using bootstrap sampling distributions. **(E–H)** The fractions of Th17 cells were determined by flow cytometry. **(E)** Plot showing the gating strategy for identifying CD8 negative T-cells as surrogates of T-helper cells. **(F)** Plot of CD8^−^ T-cells IL17 content without stimulation. **(G)** As in previous panel but with stimulation. **(H)** The fraction of Th17 cells in controls (healthy blood donors and parents) and triplets. The columns are the geometric means of the two groups. The fold-difference of the means was estimated with 95% CIs using bootstrap sampling distributions. As an in-house reference, the central 90%-prediction interval of the Th17 cell measurements from 51 healthy blood donors was [0.21%; 1.7%].

In support of our hypothesis, we found that, after IL6 stimulation of peripheral blood mononuclear cells, there was a mean 1.4-fold (95% CI: 1.1–1.7) relative increase in cells with phosphorylated STAT3 in the triplets compared to controls (two healthy blood donors and the parents, Figures 2A–D; Supplementary Table S2).

STAT3 phosphorylation is essential for the development of Th17 cells, which are important drivers of a range of autoimmune diseases (Yasuda et al., 2019). Given the increased fraction of cells with phosphorylated STAT3, we examined the triplet’s fraction of Th17 cells. We found that the triplets also had a mean 2.7-fold (95% CI: 1.8–3.9) relative increase in the fraction of Th17 cells (Figures 2E–H; Supplementary Table S2), compared to controls (six healthy blood donors and the parents).

Finally, we performed western blotting analyses of PTPRM expression in PBMCs and serum, but we were not able to detect any differences in PTPRM expression in patients versus parents/controls in our experiments (Supplementary Figures S3, S4).

Taken together, we propose that the deletion of *PTPRM* may contribute to the triplets’ autoimmune manifestations by reducing the negative regulation of STAT3 phosphorylation, thereby increasing Th17 cell fractions and promoting autoimmunity.

## 4 Discussion

We describe a unique case of monozygotic triplets concordant for developmental delay, WM lesions, autoimmune manifestations, and MPO-ANCA positivity in whom we identified a *de novo* interstitial deletion of chromosome 18p. The deletion spanned 19 protein-coding genes of which, we hypothesized *PTPRM* hemizygosity as a potential cause of their autoimmune susceptibility. In support of this hypothesis, we identified the patient cells to display increased cell levels with phosphorylated STAT3 and increased Th17 cell fractions upon stimulation.

18p deletion syndrome is a chromosomal anomaly characterized by clinical variability involving developmental delay, facial dysmorphology, and brain abnormalities. Some patients also present with autoimmune disease, which typically includes autoimmune thyroid disease and arthritis (Sebold et al., 2015; McGoey et al., 2011). This includes cases of juvenile-onset Graves’ disease (Jones and Carey, 1982; Dharmaraj and Grueters, 2006).

According to current knowledge, most reported pathogenic deletions are terminal spanning large proportions of the 18p arm, whereas interstitial 18p deletions are rare (Hasi-Zogaj et al., 2015). Smaller deletions involving fewer genes found in patients with concordant phenotypes are highly valuable for the identification of genes underlying the observed phenotype. Known disease-associated genes within the deletion included *APCDD1*, *LAMA1, NDUFV2, and PIEZO2*. Heterozygosity of a missense variant in *APCDD1* has been reported to cause hypotrichosis, likely through a dominant-negative mechanism (Shimomura et al., 2010). *LAMA1* and *NDUFV2* biallelic variants cause Poretti-Boltshauser syndrome (Aldinger et al., 2014) and mitochondrial complex I deficiency (Bénit et al., 2003), respectively. Monoallelic gain-of-function variants and biallelic loss-of-function variants in *PIEZO2* cause distal arthrogryposis (Coste et al., 2013; Chesler et al., 2016). Hemizygosity of these genes was therefore not considered to explain the triplets’ phenotype. As mentioned, the deletion also included *MTCL1* – a gene that has been associated with ataxia (Satake et al., 2017; Krygier et al., 2019), which the triplets presented with. Ataxia has also previously been reported in 18p deletion syndrome (Wester et al., 2006; Kumar et al., 2017). In mice, knockdown of MTCL1 has been shown to cause Purkinje cell degeneration and abnormal cerebellar motor coordination (Satake et al., 2017). The potential role of *MTCL1* hemizygosity, however, remains uncertain. Other genes at chromosome 18p (*GNAL* and *AFG3L2*) have previously been linked to motor dysfunction but were not included in the triplet’s deletion.

From searching the literature, we identified *PTPRM* as a putative candidate gene underlying the immune dysregulation in the triplets. Based on recent experimental studies, showing *PTPRM* knockdown (Im et al., 2020) or induced *PTPRM* methylation (Song et al., 2021) to increase STAT3 phosphorylation in cancer cells, we hypothesized *PTPRM* hemizygosity as a result of the interstitial 18p deletion in the triplets to cause increased STAT3 phosphorylation in immune cells. STAT3 activation has a central role in immune regulation which involves Th17 cell differentiation (Yasuda et al., 2019). The importance of STAT3 function in immune regulation is demonstrated by the clinical manifestations in STAT3 gain-of-function (GOF) syndrome which often include early-onset autoimmunity including endocrinopathy with type 1 diabetes mellitus and thyroid disease (Flanagan et al., 2014). In rare cases, STAT3 GOF syndrome may also present with systemic vasculitis, including cerebral and pulmonary vasculopathy (Leiding et al., 2023), which had also been suspected from radiological imaging of our patients, although not ascertained. In contrast to *STAT3* GOF variants, inactivating *STAT3* variants cause hyper-IgE syndrome which is associated with decreased Th17 cell fractions and chronic mucocutaneous candidiasis (Tangye and Puel, 2023).

In support of the hypothesis, we observed an increase in cells with phosphorylated STAT3 in the patients upon stimulation, which may indicate functional importance of *PTPRM* hemizygosity. Furthermore, we found increased Th17 cell fractions in the triplets compared to healthy controls. Th17 are proinflammatory cells characterized by their production of IL-17, which also have been associated with immune disorders (Yasuda et al., 2019) including thyroid disease (Vitales-Noyola et al., 2017) and chronic urticaria (Toubi and Vadasz, 2020) as seen in our patients. We therefore propose that *PTPRM* hemizygosity conveys susceptibility to Th17-mediated autoimmunity. This raises the question of whether the patients’ autoimmune disease could be ameliorated by IL-17A inhibition (e.g., secukinumab), which remains to be ascertained.

The findings in this study are preliminary and several limitations should be considered in the interpretation of the results. First, we could not confirm differential PTPRM expression following gene hemizygosity in our experiments. PTPRM expression in blood cells is generally considered to be low, which may have limited our ability to demonstrate an actual difference. Also, non-specific binding of the applied antibody toward the phosphatase could have impacted the results. Furthermore, from our results, we cannot conclude on the isolated effect of *PTPRM* hemizygosity on autoimmune susceptibility. Other both genetic and non-genetic factors may contribute to the concordant autoimmunity with the triplets sharing their entire genomic variation and growing up in the same household. We therefore emphasize the need for investigations of unrelated patients with *PTPRM* hemizygosity to ascertain this hypothesis and better understand the mechanism(s). Furthermore, it should be noted that not all 18p deletion syndrome patients are reported with an autoimmune disease (Hasi-Zogaj et al., 2015). However, we considered the previous reports of juvenile-onset Graves’ disease in 18p deletion patients (Sebold et al., 2015; Jones and Carey, 1982; Dharmaraj and Grueters, 2006) to support our hypothesis. Finally, no targeted interventions (i.e., IL-17 blockade) have been attempted to treat the autoimmune manifestations. A disease-modifying effect hereof would further support the proposed disease-causing impact of *PTPRM* hemizygosity through Th17-mediated autoimmune susceptibility.

In conclusion, we identified monozygotic triplets concordant for developmental delay and early-onset autoimmunity to harbor an interstitial 18p deletion including *PTPRM*–a negative regulator of STAT3. Since the triplets showed increased cellular STAT3 phosphorylation together with increased Th17 cell fractions, we propose *PTPRM* hemizygosity as a putative genetic driver of immune dysregulation in 18p deletion syndrome. This finding could have potential therapeutic implications for 18p deletion syndrome patients presenting with autoimmune disorders, warranting further studies in unrelated patients.

## Data Availability

The original contributions presented in the study are included in the article/Supplementary Material, further inquiries can be directed to the corresponding author.
