# Supplementary material for: Monozygotic triplets with juvenile-onset autoimmunity and 18p microdeletion involving PTPRM
Source: Front Genet. 2024 Sep 18;15:1437566. doi: 10.3389/fgene.2024.1437566 (PMC11445036; doi:10.3389/fgene.2024.1437566)
Supplement: Supplementary file 1 [file Table2.DOCX]

Supplementary Material

# Supplementary Methods

## Genetic analysis

### Microarray-based comparative genomic hybridization (array CGH)

For chromosomal microarray analysis, all samples were screened using the SurePrint G3 Human CGH microarray 4x180K (Agilent Technologies) with an approximate resolution of 50kb. Sample and reference genomic DNA (500 ng) were labeled with Cy5 (reference) or Cy3 (specimen) using the Sure TagComplete DNA labelling Kit (Agilent Technologies Inc.) and purified as per the manufacturer’s protocol. Labeled sample and reference DNA were pooled and 5μL human COT-1 DNA (1mg/mL), 10x blocking agent and 2x Hi-RPM hybridization buffer were added. Hybridization lasted 20-24 hours at 67 ◦C. Scanning and image acquisition were carried out using an Agilent microarray scanner and microarray image files were quantified using Agilent’s Feature Extraction software, version 10.7. Data analysis was performed using CytoGenomics, version v.5.1.2.1 (Agilent Technologies Inc.). Copy number was determined using the adm-2 algorithm and profile deviations consisting of four or more neighboring oligonucleotide probes were considered genomic aberrations.

### Whole genome sequencing

We performed whole genome sequencing (WGS) of DNA from one of the triplets (triplet A) for accurate breakpoint characterization, sequence analysis for biallelic involvement of the deleted genes, and sequence analysis of an *in silico* gene panel related to inborn errors of immunity (IEI, see below). TruSeq PCR-free DNA sample preparation kit (Illumina Inc.) was used to prepare WGS libraries followed by paired-end sequencing on a NovaSeq 6000 platform (Illumina Inc.) to a mean coverage of 30x. Initial data analysis followed a local NBA2 pipeline (Dept. of Molecular Medicine, Aarhus University Hospital) adhering to the GATK best practice recommendations. Structural variants including copy number alterations were called by Manta [1], Delly2 [2], and Lumpy [3]. Data visualization was done in VarSeq version 2.3.0 (Golden Helix) and Integrative Genomics Viewer v2.16.2.

### Variant filtration and IEI gene panel analysis

Filtration of variants identified by WGS analysis included genotype quality (GQ) ≥ 20, variant allele frequency ≥ 0.1 or missing and one of two filtration algorithms. Algorithm 1 included allele frequency in gnomAD v4.0 ≤ 0.01 or missing, homozygous count in gnomAD v4.0 < 5 or missing and one the following four criteria:

- Loss-of-function or missense variants
- Variants other than loss-of-function or missense variants with CADD (PHRED) ≥ 25
- Sequence ontology is exon_loss_variant, non_coding_exon_variant, or stop_retained_variant
- Variants other than loss-of-function or missense variants with Ada score >0.6 or predicted splicing disrupted (MaxEntScan)

Algorithm two required one of the following two criteria

- Variant class is ‘DM’ in the Human Gene Mutation Database (version 2023.3)
- Variant classified as ‘likely pathogenic’ or ‘pathogenic’ in ClinVar (2024-01-04)

Filtration of variants then included a large consensus *in silico* gene panel of 341 genes involved in cellular and innate immune dysregulation according to the Danish Society of Clinical Immunology. The gene panel included: *ACD, ACTB, ADA, ADA2, AK2, APOL1, ARHGEF1, ARPC1B, ATG4A, ATM, B2M, BCL10, BCL11B, BLM, BLOC1S6, BRCA1, BRCA2, BRIP1, C1QA, C1QB, C1QC, C1R, C1S, C2, C3, C4A, C4B, C5, C6, C7, C8A, C8B, C8G, C9, CARD11, CARD9, CCBE1, CD247, CD28, CD3D, CD3E, CD3G, CD4, CD40, CD40LG, CD46, CD55, CD59, CD8A, CDCA7, CEBPE, CFB, CFD, CFH, CFHR1, CFHR2, CFHR3, CFHR4, CFHR5, CFI, CFP, CFTR, CHD7, CHUK, CIB1, CIITA, CLCN7, CLEC7A, CLPB, COG6, COPG1, CORO1A, CRACR2A, CSF2RA, CSF2RB, CSF3R, CTC1, CTSC, CXCR2, CXCR4, CYBA, CYBB, CYBC1, DBR1, DCLRE1C, DIAPH1, DKC1, DNAJC21, DNMT3B, DOCK2, DOCK8, DOCK11, EFL1, ELANE, EPG5, ERBIN, ERCC4, ERCC6L2, EXTL3, FANCA, FANCB, FANCC, FANCD2, FANCE, FANCF, FANCG, FANCI, FANCL, FANCM, FAS, FAT4, FCGR1A, FCGR3A, FCHO1, FCN3, FERMT3, FOXN1, FPR1, G6PC3, G6PD, GATA1, GATA2, GFI1, GINS1, GJC2, HAX1, HELLS, HMOX1, HYOU1, ICOS, ICOSLG, IFIH1, IFITM3, IFNAR1, IFNAR2, IFNG, IFNGR1, IFNGR2, IKBKB, IKBKG, IKZF1, IKZF2, IKZF3, IL12B, IL12RB1, IL12RB2, IL17F, IL17RA, IL17RC, IL18BP, IL21, IL21R, IL23R, IL2RG, IL6R, IL6ST, IL7R, IRAK1, IRAK4, IRF3, IRF4, IRF7, IRF8, IRF9, ISG15, ITGB2, ITK, ITPKB, JAGN1, JAK1, JAK3, KDM6A, KMT2A, KMT2D, KRAS, LAMTOR2, LAT, LCK, LCP2, LIG1, LIG4, MAD2L2, MALT1, MAN2B1, MAN2B2, MAP1LC3B2, MAP3K14, MAPK8, MASP2, MCM10, MCM3AP, MCM4, MECOM, MPO, MRTFA, MSN, MTHFD1, MYD88, MYSM1, NBAS, NBN, NCF1, NCF2, NCF4, NCKAP1L, NCSTN, NFE2L2, NFKBIA, NHEJ1, NHP2, NLRP3, NOP10, NOS2, NRAS, NSMCE3, ORAI1, OSTM1, PALB2, PARN, PAX1, PCCA, PCCB, PGM3, PLEKHM1, PMM2, PMS2, PNP, POLD1, POLD2, POLE, POLE2, POLR3A, POLR3C, POLR3F, PRKDC, PRPS1, PSEN1, PSENEN, PTPRC, RAC2, RAD51, RAD51C, RAG1, RAG2, RANBP2, RBCK1, REL, RELA, RELB, RFWD3, RFX5, RFXANK, RFXAP, RHOH, RIPK1, RMRP, RNF168, RNF31, RNU4ATAC, RORC, RPSA, RTEL1, SAMD9, SAMD9L, SASH3, SBDS, SEMA3E, SERAC1, SERPING1, SKIV2L, SLC35C1, SLC37A4, SLC46A1, SLX4, SMARCAL1, SMARCD2, SNORA31, SNX10, SP110, SPINK5, SPPL2A, SRP54, SRP72, STAT1, STAT2, STAT3, STAT4, STAT5B, STIM1, STK4, STN1, SYK, TAFAZZIN, TAP1, TAP2, TAPBP, TBX1, TBX21, TCIRG1, TCN2, TERC, TERT, TET2, TFRC, TGFBR1, TGFBR2, THBD, TICAM1, TINF2, TIRAP, TLR3, TLR7, TLR8, TMC6, TMC8, TNFRSF11A, TNFRSF4, TNFSF11, TOM1, TP53, TRAC, TRAF3, TRAF3IP2, TTC37, TTC7A, TYK2, UBE2T, UNC119, UNC93B1, USB1, VPS13B, VPS45, WAS, WDR1, WIPF1, WRAP53, XRCC2, ZAP70, ZBTB24, ZNF341*, and *ZNFX1*.

## Functional assays

### STAT3 phosphorylation assay

Heparinized peripheral blood (200 µL) from patients, parents, and two healthy blood donor controls was stimulated for 15 minutes at +37°C with recombinant human IL-6 (BD Pharmingen, cat. 55071) at a final concentration of 100 ng/mL. Cells were lysed, fixed, permeabilised and stained according to standard BD protocol for intracellular antibody staining based on Phosflow Lyse/Fix buffer (BD Biosciences cat. 558049), Phosflow Perm Buffer III (BD Biosciences cat. 558050) and Stain Buffer (BD Biosciences cat. 554656). Specifically, cells were stained with a phosphorylation-specific PE-conjugated mouse-anti-human Stat3 (pY705) antibody (BD Biosciences cat. 562072 – Clone 4/P-STAT3) and an APC-conjugated mouse-anti-human CD45 (BD Biosciences cat. 340910). Controls included concurrent stains of non-stimulated blood, and healthy blood donor controls, as well as technical controls (compensation controls, unstained controls, QC beads). Cells were analyzed on an ACEA/Agilent NovoCyte 3000 flow cytometer in standard 3-laser configuration to identify relevant cell populations and quantify relevant fluorescence signals.

### Th1, Th2, Th17 assay

Briefly, peripheral blood mononuclear cells (PBMCs) were isolated by density centrifugation (Leucosep™ tubes, Greiner) of heparinized peripheral blood samples from patients, parents, and six healthy blood donor controls. After isolation, cells were resuspended in complete RPMI 1640 culture medium w. 10% human serum and rested overnight by incubation at +37°C, 5% CO2. The following morning, cells were stimulated for 5 hours (at +37°C, 5% CO2) with 50 ng/mL PMA (VWR cat. A0903.0005) and 1.3 μM ionomycin (Sigma-Aldrich cat. I3909) in the presence of BD GolgiStop™ solution. After stimulation, cells were permeabilized and stained for CD4 and intracellular IFN-γ, IL-4, and IL-17A with the Human Th1/Th2/Th17 Phenotyping Kit (BD Biosciences cat. 560751) according to the recommended protocol. Cells were further stained with BV785-conjugated mouse-anti-human CD8a antibody (Nordic BioSite cat. 301046) and APC-Cy7-conjugated mouse-anti-human CD3 antibody (Nordic BioSite cat. 344818) for enhanced resolution of T-cell subsets. Controls included concurrent stains of non-stimulated cells (both for patients and healthy donors) as well as technical controls (compensation controls, unstained controls, QC beads). Cells were analyzed on an ACEA/Agilent NovoCyte Quanteon flow cytometer in standard 4-laser configuration to identify relevant cell populations and quantify fluorescence signals from all three cytokine stains simultaneously. Th17 cells were defined as CD3+ CD8a- IL-17A+.

### Regulatory T cells (FoxP3) assay

Heparinized peripheral blood (50 µL) was stained for membrane expression of CD4, CD127, CD25, and CD45RA by titrated amounts of relevant antibodies (in BD Brilliant Stain Buffer) and incubation for 30 minutes at room temperature. Specifically, cells were stained with FITC-conjugated mouse-anti-human CD4 (BD Biosciences cat. 345768), PE-Cy7-conjugated mouse-anti-human CD127 (BD Biosciences cat. 560882), BV421-conjugated mouse-anti-human CD25 (BD Biosciences cat. 562442), and BV605-conjugated mouse-anti-human CD45RA (BD Biosciences cat. 532886). Cells were then fixed, permeabilized (PerFix-nc Kit Beckman-Coulter cat. B31167) and stained intracellularly by addition of AF647-conjugated mouse-anti-human FoxP3 antibody (Nordic BioSite cat. 320214). Controls included concurrent stains of healthy blood donor controls, as well as technical controls (compensation controls, QC beads). Cells were analysed on an ACEA/Agilent NovoCyte 3000 flow cytometer in standard 3-laser configuration to identify regulatory T cells (CD4+ CD25+ FoxP3+ CD127-) as a percentage of total CD4+ T cells.

### Immunophenotyping assay

Both relative and absolute concentrations of key immunological cellular subsets were determined in heparinized peripheral blood (200 µL) with three distinct panels of antibodies: a broad lineage-panel, a T-cell specific panel, and a B-cell specific panel. For relative concentrations, blood samples were lysed and washed during the staining procedure (using BD FACS Lysing Solution, cat. 349202), whereas absolute concentrations were determined on the basis of a lyse-no-wash protocol of the lineage-panel to avoid cell loss (by ammonium chloride lysing solution (VWR cat. AMPQ44033.0500)). Absolute concentrations of T- or B-cell subsets were then calculated from the relative fraction of the subset relative to the total T- or B-cell populations. All antibody stains were at room temperature for 30 minutes using titrated amounts of antibody and BD Brilliant Stain Buffer (BD Pharmingen cat. 563794), either directly in the blood sample (for absolute concentrations) or in washed blood (washed in PBS w. 0.1% Bovine Serum Albumin) (for relative concentrations). The antibodies used in the three panels were: Lineage-panel: CD45 [APC-H7] (BD Pharmingen cat. 560178), CD16 [APC] (BD Pharmingen cat. 561304), CD56 [BV421] (BD Pharmingen cat. 562750), CD14 [PE-Cy7] (BioLegend cat. 301814), CD3 [FITC] (BD Pharmingen cat. 555332), and CD19 [PE] (BD Pharmingen cat. 555413) ; T-cell panel: CD3 [APC-Cy7] (BioLegend cat. 344818), CD8a [BV785] (BioLegend cat. 301046), CD27 [BV421] (BioLegend cat. 302824), CD4 [BV570] (BioLegend cat. 300534), CD197 (CCR7) [PE] (BioLegend cat. 353204), CD45RA [APC] (BioLegend cat. 304112), TCR γδ [FITC] (BioLegend cat. 331208), and HLA-DR [PE-Cy7] (BioLegend cat. 331208) ; B-cell panel: CD21 [PE-Cy7] (BioLegend cat. 354912), Kappa-LC [APC] (BioLegend cat. 316510), IgD [PE] (Thermo Fisher cat.12-9868-42), CD27 [VioBright FITC] (Miltenyi Biotec cat. 130-113-634), CD19 [SuperBright 600] (Thermo Fisher cat. 63-0198-42), CD38 [BV421] (BioLegend cat. 303526), and CD20 [BV785] (BioLegend cat. 302356). Controls included concurrent stains of healthy blood donor controls, as well as technical controls (compensation controls, QC beads). Cells were analyzed on an ACEA/Agilent NovoCyte 3000 flow cytometer in standard 3-laser configuration to identify the following immunological subsets on the basis of antibody staining patterns: T cells, B cells, NK cells, NKT cells, monocytes, and granulocytes. T cells were further identified as Helper T cells (CD4+) and Cytotoxic T cells (CD8+), (each subclassified as Naïve, Central memory, or Effector memory) and assessed with respect to activation status (HLA-DR) and TCR γδ expression. B cells were further identified as Transitional, Naïve, Class-switched memory, Plamablasts, and Marginal-zone-like B cells; and classified according to CD21 expression level.

### Western blotting of PTPRM expression

Cells were harvested for western blotting by removal of growth medium and washing with PBS. Cells were lysed in PierceTM RIPA Buffer (Thermo Fisher Scientific #89901) with cOmplete Mini EDTA-free Protease inhibitor Cocktail (Roche Diagnostics #5892953001) containing 0.2 µL/mL benzonase (Sigma Aldrich #E1014). Sample protein amounts were normalized using Pierce BCA Protein Assay Kit (Thermo Fisher Scientific #23225). Since serum is cell-free, no lysis was necessary and serum was diluted 1:100 in 1x tris buffered saline (TBS, Fisher Scientific #BP2471-100) without protein determination and otherwise handled the same as cell lysates.

Samples were denatured for 5 minutes at 95 °C with addition of 50 mM dithiothreitol and 4x Laemmli Buffer (Bio-Rad #1610747). Sample protein contents were separated by SDS gel electrophoresis using a precast gel (Bio-Rad #3450033) and transferred to PVDF membrane (Bio-Rad # #1704156) using the Transfer Blot Turbo System (Bio-Rad). Following transfer to the membrane, the blot was washed briefly in 1x TBS containing 0.1% Tween (TBS-T, Sigma Aldrich #P1379) and then blocked for at least 1h in the same solution with addition of 5% skim milk powder (Sigma Aldrich #70166-500G). Following blocking blots were incubated overnight with primary antibody diluted 1:1000 in 1x TBS-T with 5% bovine serum albumin (Sigma Aldrich #A7906-100G) against PTPRM (R&D Systems #MAB4446-SP) or Vinculin (Cell Signaling Technology #CST-13901S). The following day the blots were washed briefly in 1x TBS-T and incubated for 1 hour with secondary antibody against anti-mouse (Jackson ImmunoResearch #715-036-150) or anti-rabbit (Jackson ImmunoResearch #711-035-152) diluted 1:10000 in 1x TBS-T with 5% skim milk at room temperature. After washing excess secondary antibody away, blots were developed using ClarityTM Western ECL Substrate (Bio-Rad #1705061) or ClarityTM Max Western ECL Substrate (Bio-Rad #1705062) in BioRad ChemiDoc Imaging System.

Densitometry analysis of the immunoblot band intensity was performed using the Image J software. The selection area for intensity measurement was used for all measurements in the respective immune blot to avoid bias. In some cases, the densitometry measurement of PTPRM was normalized to the densitometry measurement of the samples' corresponding loading control to provide a normalized relative protein amount.

# Supplementary Figures and Tables

## Supplementary Tables

### Supplementary Table S1

See separate spreadsheet.

### Supplementary Table S2

Supplementary Table S2 shows the results of the immunological examination of the triplets, their parents and health control samples (blood donors).

| **Supplementary Table S2.** Immunological parameters in the patients, parents and blood donor controls. | | | | | | | | | | | | |
| --- | --- | --- | --- | --- | --- | --- | --- | --- | --- | --- | --- | --- |
|  | F | M | T1 | T2 | T3 | C1 | C2 | C3 | C4 | C5 | C6 | Reference (95%) interval |
| Lymphocytes/µL | 1880 | 2131 | 1177 | 1307 | 1107 |  |  |  |  |  |  | 1300; 3500 |
| STAT3p+, % of lymphocytes | 41 | 37 | 58 | 70 | 62 | 49 | 59 |  |  |  |  | N/A |
| T-cells/µL | 1575 | 1227 | 942 | 1088 | 920 |  |  |  |  |  |  | 581; 1895 |
| CD4+CD8-, % | 62 | 55 | 73 | 78 | 73 |  |  |  |  |  |  | 44; 80 |
| Naive, % | 21 | 59 | 33 | 38 | 37 |  |  |  |  |  |  | 25; 73 |
| Th17 of CD3+CD8-, % (stimulated) | 0.4 | 0.3 | 1.3 | 1.00 | 1.00 | 0.2 | 0.2 | 0.6 | 0.5 | 0.7 | 0.8 | 0.21%;1.7% * |
| Th17 of CD3+CD8-, % (unstimulated) | 0.06 | 0.11 | 0.09 | 0.10 | 0.10 | 0.12 | 0.09 | 0.10 | 0.09 | 0.08 | 0.14 |  |
| Th1 of CD3+CD8-, % | 18 | 12 | 26 | 21 | 22 | 8.5 | 12 | 14 | 16 | 18 | 18 | 8.4%;44% * |
| Th2 of CD3+CD8-, % | 0.8 | 1.6 | 0.4 | 0.5 | 0.4 | 0 | 1.9 | 2.2 | 1.2 | 0 | 1.7 | 0.1%;3.0% * |
| Tregs of CD4+CD3+, % | 4.9 | 7.0 | 6.9 | 7.9 | 7.8 |  |  |  |  |  |  | 4.0; 10 |
| CD4-CD8+, % | 31 | 41 | 23 | 19 | 24 |  |  |  |  |  |  | 18;52 |
| Naive, % | 8 | 23 | 31 | 59 | 35 |  |  |  |  |  |  | 6.8; 73 |
| TCRg/d+, % | 6 | 3 | 2 | 2 | 1 |  |  |  |  |  |  | 0; 10 |
| HLA-DR+, % | 16 | 19 | 9 | 8 | 9 |  |  |  |  |  |  | 0; 10 |
| B-cells/µL | 234 | 319 | 113 | 117 | 96 |  |  |  |  |  |  | 70; 272 |
| Transitional, % | 1,5 | 0.4 | 1.7 | 1.9 | 0.2 |  |  |  |  |  |  | 1.5; 10 |
| Naive, % | 69 | 66 | 73 | 75 | 70 |  |  |  |  |  |  | 41; 79 |
| Class-switched memory, % | 3 | 6 | 3 | 2 | 3 |  |  |  |  |  |  | 4.8; 28 |
| MZ-like, % | 3.8 | 14 | 3 | 2 | 4.6 |  |  |  |  |  |  | 4.7; 39 |
| Plasmablasts, % | 0.7 | 0.4 | 1.3 | 1.3 | 1.2 |  |  |  |  |  |  | 0.1; 0.9 |
| NK-cells/µL | 71 | 585 | 122 | 102 | 91 |  |  |  |  |  |  | 52; 639 |
| Abbreviations: C, control; F, father; M, mother; T, triplet. * Central 90%-prediction interval based on measurements from 51 healthy blood donors using this assay | | | | | | | | | | | | |

## Supplementary Figures

### Supplementary Figure S1


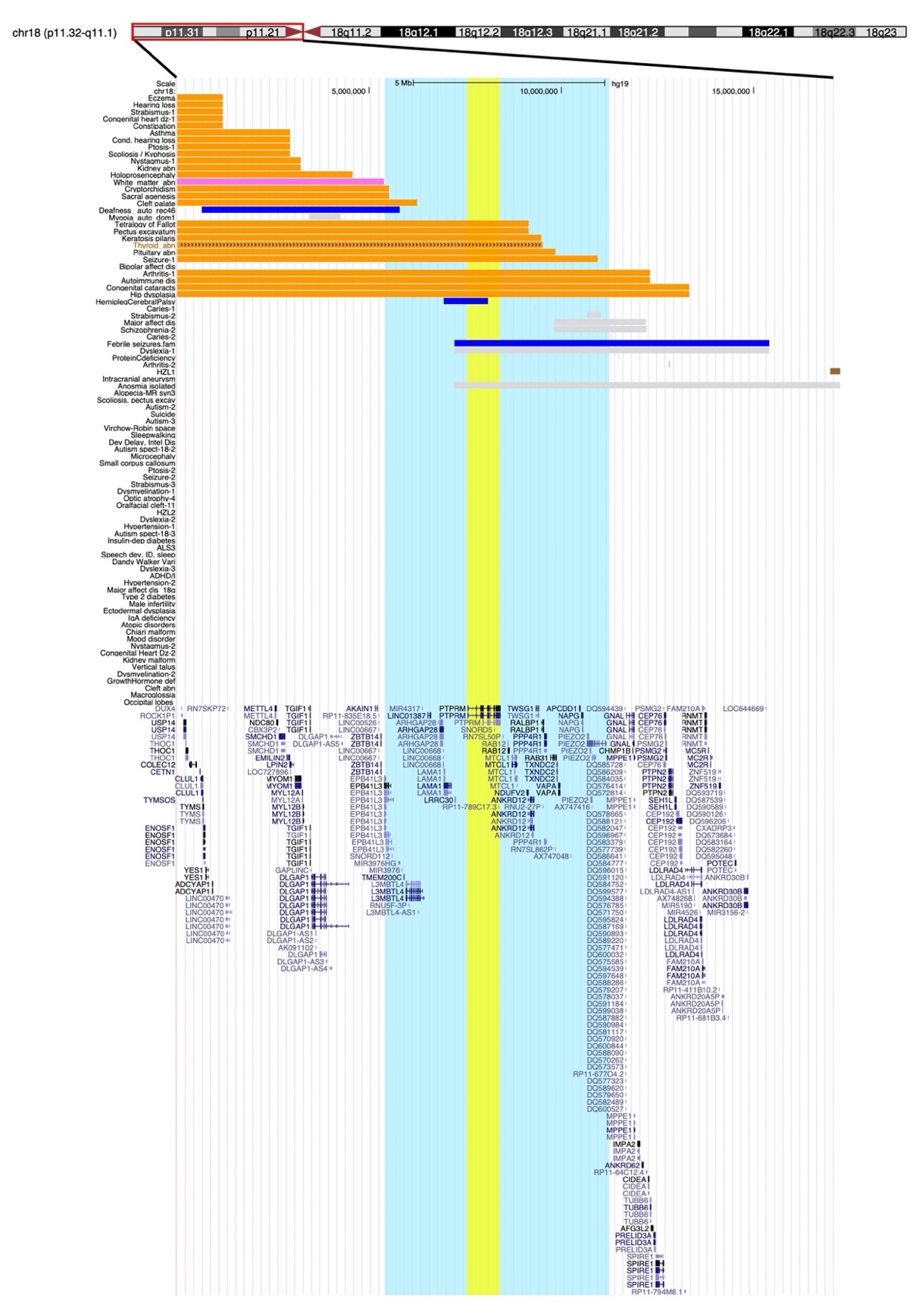


**Supplementary Figure S1.** The 18p deletion (light blue), *PTPRM* (yellow), and the phenotype map of 18p deletions according to the Chromosome 18 Gene Dosage Map [4,5]. The deletion and *PTPRM* are included in the ‘autoimmune disease’ critical region with *PTPRM* also included in the ‘thyroid abnormality’ critical region (orange bars).

### Supplementary Figure S2


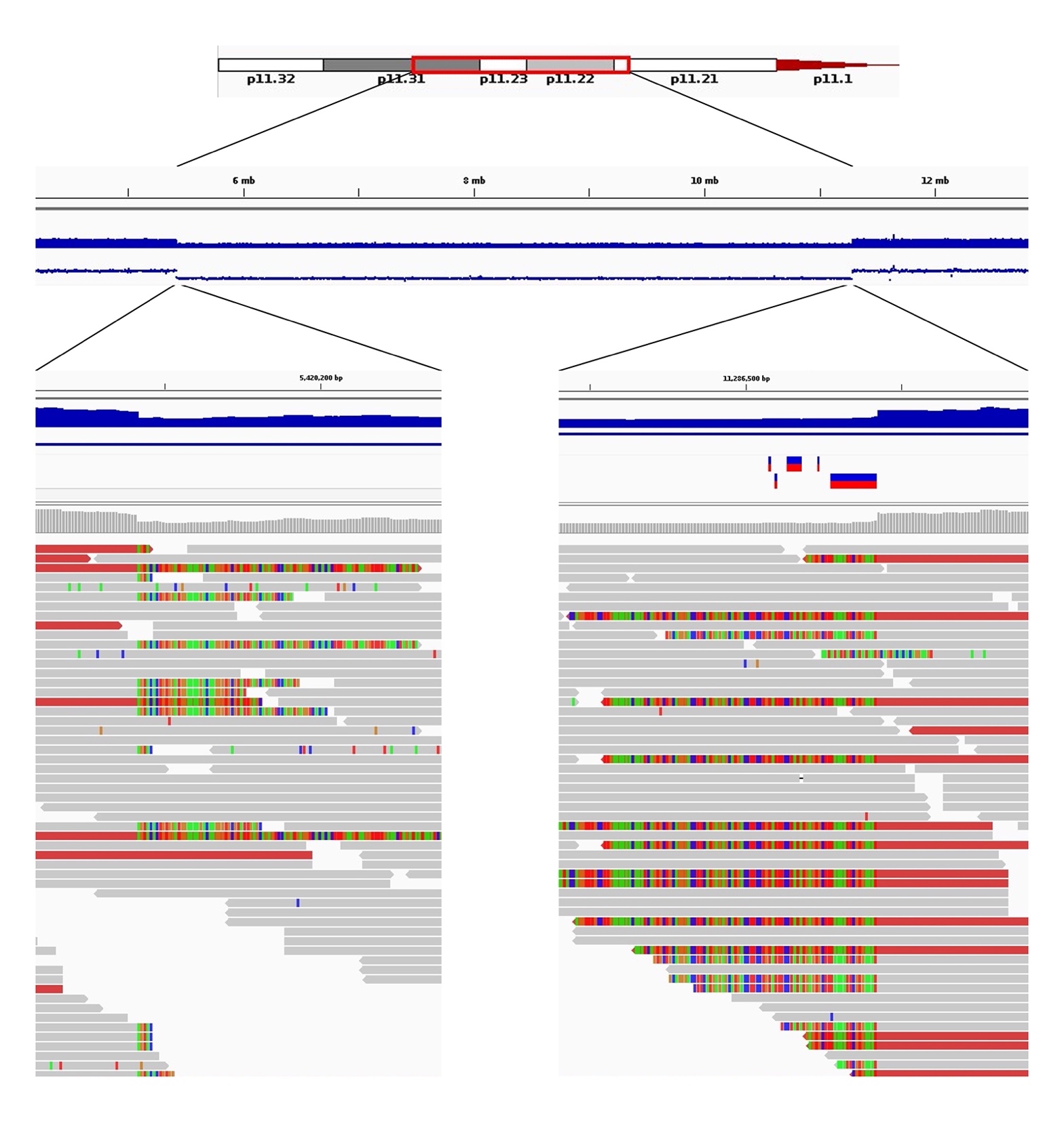


**Supplementary Figure S2.** Visualization in Integrative Genomics Viewer (IGV) of the 18p microdeletion, NC_000018.10: g.5420142_11286542del, and its breakpoints from whole-genome sequencing analysis. The decrease in read depth (height of the blue bar) reveals the location and size of the deletion. Multicolored reads indicate “soft-clipping”/misalignment with the reference genome, which reveals the accurate breakpoint locations of the deletion.

### Supplementary Figure S3


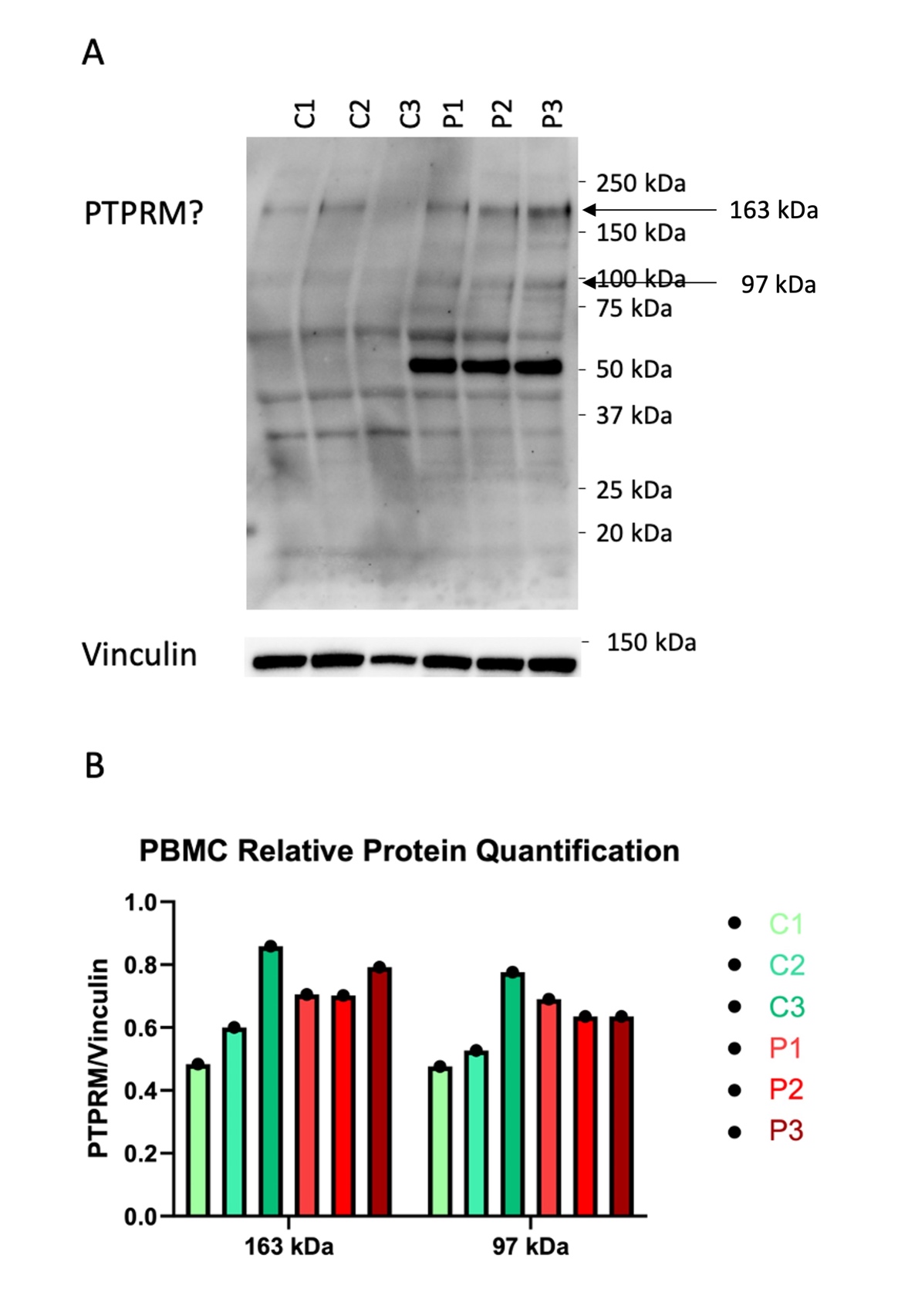


**Supplementary Figure S3.** Western blot analysis of PBMC protein lysates from the patients (P1-P3) and controls (C1-C3) using PTPRM antibody (R&D Systems #MAB4446-SP) and vinculin. A. Two bands at 163 kDa and 97 kDa were selected for quantification. B. PTPRM expression (the two candidate bands) relative to vinculin expression across samples.

### Supplementary Figure S4

**
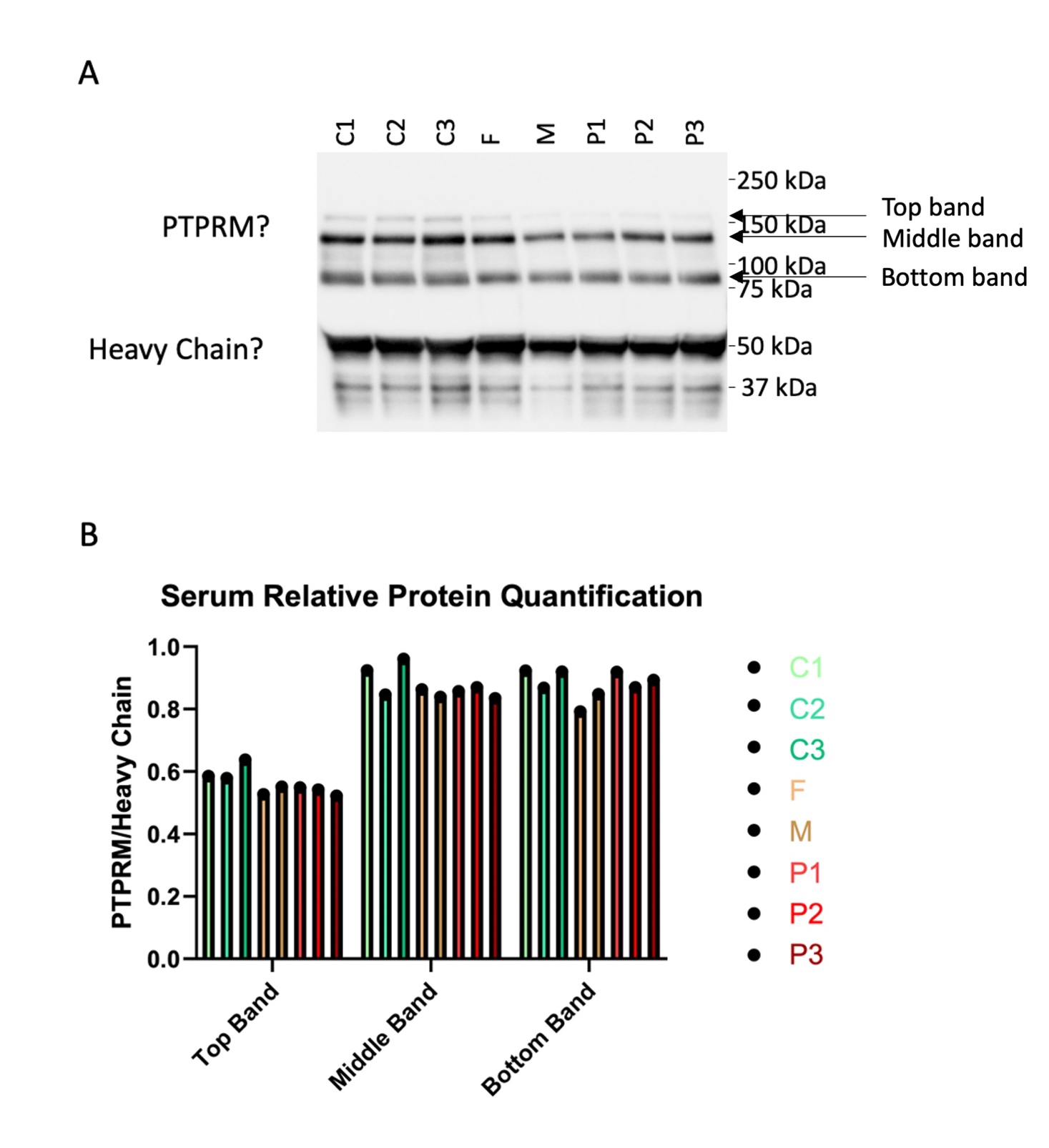
**

**Supplementary Figure S4.** Whole western blot of 1% serum protein from the triplets (P1-P3), parents (M+F) and healthy controls (C1-C3) using PTPRM antibody (R&D Systems #MAB4446-SP). A. Three bands were identified and selected for quantification. The strong band around 50kDa was interpreted as heavy chain. B. PTPRM expression (the three candidate bands) relative to heavy chain expression across samples.

# Supplementary References

1. Chen X, Schulz-Trieglaff O, Shaw R, *et al.* Manta: rapid detection of structural variants and indels for germline and cancer sequencing applications. *Bioinformatics* 2016;**32**:1220–2. doi:10.1093/bioinformatics/btv710

2. Rausch T, Zichner T, Schlattl A, *et al.* DELLY: structural variant discovery by integrated paired-end and split-read analysis. *Bioinformatics* 2012;**28**:i333–9. doi:10.1093/bioinformatics/bts378

3. Layer RM, Chiang C, Quinlan AR, *et al.* LUMPY: a probabilistic framework for structural variant discovery. *Genome Biol* 2014;**15**:R84. doi:10.1186/gb-2014-15-6-r84

4. Cody JD, Heard P, Rupert D, *et al.* Chromosome 18 gene dosage map 2.0. *Hum Genet* 2018;**137**:961–70. doi:10.1007/s00439-018-1960-6

5. Chromosome 18 Clinical Research Center: Chromosome 18 Gene Dosage Map. https://wp.uthscsa.edu/chrome-18/research/ (accessed 7 Sep 2023).
